# Thoracic spine manipulation for the management of mechanical neck pain: A systematic review and meta-analysis

**DOI:** 10.1371/journal.pone.0211877

**Published:** 2019-02-13

**Authors:** Michael Masaracchio, Kaitlin Kirker, Rebecca States, William J. Hanney, Xinliang Liu, Morey Kolber

**Affiliations:** 1 Department of Physical Therapy, Long Island University, Brooklyn, New York, United States of America; 2 Department of Health Professions, University of Central Florida, Orlando, Florida, United States of America; 3 Department of Health Management and Informatics, University of Central Florida, Orlando, Florida, United States of America; 4 Department of Physical Therapy, Nova Southeastern University, Fort Lauderdale, Florida, United States of America; Federal Joint Committee, GERMANY

## Abstract

**Objective:**

To investigate the role of thoracic spine manipulation (TSM) on pain and disability in the management of mechanical neck pain (MNP).

**Data sources:**

Electronic databases PubMed, CINAHL, Pedro, Embase, AMED, the Cochrane Library, and clinicaltrials.gov were searched in January 2018.

**Study selection:**

Eligible studies were completed RCTs, written in English, had at least 2 groups with one group receiving TSM, had at least one measure of pain or disability, and included patients with MNP of any duration. The search identified 1717 potential articles, with 14 studies meeting inclusion criteria.

**Study appraisal and synthesis methods:**

Methodological quality was evaluated independently by two authors using the guidelines published by the Cochrane Collaboration. Pooled analyses were analyzed using a random-effects model with inverse variance methods to calculate mean differences (MD) and 95% confidence intervals for pain (VAS 0-100mm, NPRS 0-10pts; 0 = no pain) and disability (NDI and NPQ 0–100%; 0 = no disability).

**Results:**

Across the included studies, there was increased risk of bias for inadequate provider and participant blinding. The GRADE approach demonstrated an overall level of evidence ranging from very low to moderate. Meta-analysis that compared TSM to thoracic or cervical mobilization revealed a significant effect favoring the TSM group for pain (MD -13.63; 95% CI: -21.79, -5.46) and disability (MD -9.93; 95% CI: -14.38, -5.48). Meta-analysis that compared TSM to standard care revealed a significant effect favoring the TSM group for pain (MD -13.21; 95% CI: -21.87, -4.55) and disability (MD -11.36; 95% CI: -18.93, -3.78) at short-term follow-up, and a significant effect for disability (MD -4.75; 95% CI: -6.54, -2.95) at long-term follow-up. Meta-analysis that compared TSM to cervical spine manipulation revealed a non-significant effect (MD 3.43; 95% CI: -7.26, 14.11) for pain without a distinction between immediate and short-term follow-up.

**Limitations:**

The greatest limitation in this systematic review was the heterogeneity among the studies making it difficult to assess the true clinical benefit, as well as the overall level of quality of evidence.

**Conclusions:**

TSM has been shown to be more beneficial than thoracic mobilization, cervical mobilization, and standard care in the short-term, but no better than cervical manipulation or placebo thoracic spine manipulation to improve pain and disability.

**Trial registration:**

PROSPERO CRD42017068287

## Introduction

Neck pain is prevalent in the general population, often leading to physical impairments and disability. In 2010, Hoy et al [1] conducted an international systematic review on the epidemiology of activity-limiting neck pain, reporting a one-year incidence between 10.4% and 21.3%, with a one-year remission rate between 33% and 65%. An update to the 2016 Global Burden of Disease, Injuries, and Risk Factors ranked neck pain 11^th^ overall in global cause of disability-adjusted life years [2]. Often, neck pain is mechanical in nature, and a specific pathoanatomical cause is usually unidentifiable in clinical practice. While the definition of mechanical neck pain (MNP) varies in the literature, it can be defined as pain located in the cervical spine, including the cervicothoracic junction, which is exacerbated with cervical motion, sustained postures, and/or palpation of the cervical musculature [3–6].

Conservative treatment of MNP, often includes various interventions, such as education, modalities, therapeutic exercises, non-thrust manipulation (mobilization), and thrust manipulation [7]. A recent clinical practice guideline (CPG) published in 2017 from the Orthopedic Section of the American Physical Therapy Association summarized the effectiveness of these interventions and provided overall recommendations on their clinical benefit [8]. In addition, this updated CPG separated each intervention and its overall level of recommendation based on the chronicity of symptom duration (acute, subacute, and chronic), which was lacking from the 2008 CPG on neck pain. Manual therapy to the cervical and thoracic spine in the form of mobilization and manipulation were reported to have a moderate or weak level of evidence for any symptom duration with recommendations for continued research.

Numerous clinical trials have attempted to compare and contrast mobilization and manipulation to each other and to other common interventions provided by physical therapists in the management of MNP [3, 5, 7–18]. While research on the management of MNP continues to expand, firm conclusions on which manual therapy techniques to perform, to which regions of the spine, and in what cohort of patients remain elusive. Due to perceived, albeit low risks associated with manipulation of the cervical spine [19], some research has focused on whether manipulation targeting the thoracic spine can be effective for the management of MNP, with several studies showing promising results [4, 7, 11, 13, 17, 20–22].

Several theories have been proposed to explain the mechanisms of manual therapy effects in reducing MNP. One model by Bialosky et al, suggests that a mechanical stimulus causes a cascade of mechanical and neurophysiological changes within the neuromuscular system [23]. These changes include decreased inflammatory mediators, increased serum serotonin and other endorphin mediators, decreased hypoalgesia, and decreased activation of supraspinal structures [23], any of which may lead to reduced pain and increased function. In addition, regional interdependence suggests that certain dysfunctions in a particular region of the musculoskeletal system may be effectively managed by applying interventions at remote or adjacent sites [7, 8, 24]. This is likely in the spine, as both biomechanical links and pain referral patterns between the lower cervical and upper thoracic spine have been reported in the literature [25–28].

Based on the model of regional interdependence and neurophysiological changes associated with manual therapy, several randomized controlled trials (RCTs) [7, 20, 22, 29, 30] addressing the management of MNP have directed interventions to the thoracic spine. One intervention, thoracic spine manipulation (TSM), continues to draw clinical and research attention. Three previous studies [29–31] have compared TSM to mobilization with outcomes favoring the group that received TSM. Several other RCTs [3, 5, 7, 13, 15, 32] have compared TSM to other interventions in the management of MNP, demonstrating improved outcomes with the inclusion of TSM. While moderate evidence exists for the beneficial effects of TSM, a recent CPG has determined varying levels of overall evidence for this particular intervention based on the length of current symptom duration [8].

To date, three previous systematic reviews have compared TSM to other interventions in the management of MNP [4, 33, 34]. In 2011, Cross et al [4], performed the first systematic review, which included six RCTs that examined the effect of TSM on pain, range of motion, and self-reported function using between group mean differences and effect sizes for pre-treatment to post-treatment change scores using *Cohen’s d* formula. While some form of statistical analysis was conducted by Cross et al [4], formal meta-analysis was not performed, therefore providing no overall pooled effect size for treatment effectiveness. In addition, since the publication of this systematic review, 10 additional RCTs have been published examining the role of TSM in the management of MNP.

More recently, Huisman et al [33] (2013) and Young et al [34] (2014) conducted systematic reviews on the effect of TSM in patients with MNP. While both of these reviews demonstrated positive outcomes on pain and disability from TSM, neither of them conducted meta-analysis due to the heterogeneity of interventions present in the comparison/control groups. Moreover, since the publication of Young et al [34], an additional five RCTs have been conducted. When the most recent review was conducted [34], it aimed to compare TSM to thoracic mobilization, but only one study was available [31]. Two additional manuscripts [29, 30] comparing TSM to thoracic mobilization have since been published. One further consideration among all three previous systematic reviews was the use of the PEDro scale. While the PEDro scale has previously been accepted as a sufficient tool to assess the risk of bias in RCTs, a 2015 update provided by the Cochrane Collaboration recommends using the Cochrane Collaboration Risk of Bias Tool when assessing RCTs [35].

Given the substantial increase in peer-reviewed studies, along with methodological limitations of the previous reviews, additional research and statistical analysis is warranted. Thus, the purpose of this systematic review with meta-analysis and formal grading of evidence was to assess the role of TSM compared to other physical therapy interventions on pain and disability in the management of MNP.

## Methods

### Protocol and registration

This systematic review and meta-analysis was registered in the PROSPERO database (CRD42017068287) and conducted according to the Preferred Reporting Items for Systematic Reviews and Meta-Analyses (PRISMA) guidelines [36] (Appendix A).

### Protocol changes

Several protocol changes were made after the initial registration in the PROSPERO database. While subgroup analyses were not established a priori, further evaluation of the data revealed the need for a subsequent subgroup analysis. In addition, it was intended that standard mean differences (SMD) would be used in the analysis of data. However, in order to improve readability and facilitate implementation of the results into clinical practice, the authors decided to calculate mean differences (MD) so that minimal clinical important differences (MCID) of the primary outcome measures could be discussed in relation to patient improvement. Furthermore, the authors provided an overall level of evidence using the Grading of Recommendations Assessment, Development, and Evaluation (GRADE) scale, and included an assessment of publication bias to further assess all potential sources of bias in this systematic review.

### Inclusion criteria

To be included in this systematic review, an article needed to meet the following inclusion criteria: (1) completed RCT; (2) at least 2 groups with one group receiving TSM; (3) at least one measure of pain or disability; (4) included patients with MNP (any duration acceptable); (5) written in the English language.

### Exclusion criteria

Case studies, case series, RCTs that did not specifically test the effects of TSM, and RCTs that did not have pain or disability as an outcome measure were excluded. In addition, studies involving patients with cervical radiculopathy signs and symptoms consistent with nerve root involvement were excluded.

### Search strategy

An electronic search was conducted by a health sciences librarian in January 2018 using the databases PubMed, CINAHL (EBOSCO Host), Pedro, AMED (Ovid), Embase, the Cochrane Library, and clinicaltrials.gov for all relevant articles from 2000-present. The authors included clinicaltrials.gov in an attempt to capture grey literature (completed RCTs) that may not have been published due to non-significant findings. It was decided to use the year 2000 as a cut off because a preliminary search conducted by the authors did not reveal any substantial literature on this topic prior to the year 2000.

Key words were used independently and in combination including mechanical neck pain, neck pain, thoracic manipulation, manipulation, mobilization, thoracic thrust manipulation, cervical manipulation, cervical mobilization, spinal mobilization, spinal manipulation, spinal manipulative therapy, and manual therapy (Table 1). The goal behind the search strategy was to identify all potential RCTs that assessed the role of TSM in the management of individuals with MNP. After the computerized search was completed, reference lists of all selected articles were searched by hand by two separate authors (MM and KK) to identify additional related articles. Each author (MM and KK) examined all titles and abstracts to determine initial study eligibility. Full text articles were then re-evaluated for specific inclusion criterion by MM and KK. A third author (RAS) determined final eligibility when a discrepancy exist.

**Table 1:** Literature search strategy.

Database	Search Strategy
PubMed	"cervical manipulation" OR “cervical manipulations” OR "cervical mobilization" OR “cervical mobilizations” OR “cervical mobilisation" OR "thoracic manipulation" OR "thoracic manipulations" OR "thoracic mobilization" OR "manual therapy" [tw] OR "manual therapies" OR "spinal mobilization" OR "spinal mobilizations" OR "spinal mobilisation" OR "spinal manipulation" OR "spinal manipulations" OR "spine manipulation" OR "spine manipulations" OR "spine mobilization" OR "spine mobilisation" OR "manipulative therapy" OR "manipulative therapies" OR "thrust manipulation" OR “thrust manipulations” OR "orthopedic manipulation" OR “orthopedic manipulations” OR "mobilisation therapy" OR "mobilization therapy" OR “mobilization therapies” OR "manipulation therapy" OR “manipulation therapies” OR "joint mobilization" OR “joint manipulation” OR “joint manipulations” OR “joint mobilizations” OR “joint mobilisation” OR “joint mobilisations” OR spinal manipulation [mh] OR orthopedic manipulation [mh] OR manipulation [ti] OR manipulations [ti] OR mobilization [ti] OR mobilizations [ti] OR mobilisation [ti] OR mobilisations [ti] AND “neck pain” OR “cervical spine pain” OR neck pain [mh] AND (randomized controlled trial[pt] OR controlled clinical trial[pt] OR randomized[tiab] OR placebo[tiab] OR drug therapy[sh] OR randomly[tiab] OR trial[tiab] OR groups[tiab] NOT (animals [mh] NOT humans [mh]))
EMBASE	'spine manipulation'/de OR 'joint mobilization'/de OR 'orthopedic manipulation'/de OR 'manual therapy':ab,ti OR ‘manual therapies’:ab,ti OR 'manipulation therapy’ OR ‘manipulation therapies’ OR ‘mobilisation therapy’ OR ‘mobilisation therapies’ OR ‘mobilization therapy’ OR ‘mobilization therapies’ OR ‘manipulative therapy’:ab,ti OR ‘manipulative therapies’ OR (joint OR spinal OR spine OR thoracic OR cervical OR orthopedic OR orthopaedic OR thrust) NEAR/4(manipulation* OR mobilization* OR mobilisation* OR manipulative) AND 'neck pain'/de OR 'neck pain' OR 'cervical spine pain' AND 'crossover procedure':de OR 'double-blind procedure':de OR 'randomized controlled trial':de OR 'single-blind procedure':de OR (random* OR factorial* OR crossover* OR cross NEXT/1 over* OR placebo* OR doubl* NEAR/1 blind* OR singl* NEAR/1 blind* OR assign* OR allocat* OR volunteer*):de,ab,ti
CINAHL (EBSCO)	Joint OR spinal OR spine OR thoracic OR cervical OR orthopedic OR orthopaedic OR thrust) N4 (manipulation* OR mobilization* OR mobilisation* OR manipulative) OR "manual therapy" OR "manual therapies" OR "manipulative therapy" OR "manipulative therapies" OR “manipulation therapy” OR “manipulation therapies” OR “mobilization therapy” OR “mobilization therapies” OR “mobilisation therapy” OR “mobilisation therapies” OR MH joint mobilization OR MH manipulation, orthopedic OR MH manual therapy AND MH neck pain OR "neck pain" OR "cervical spine pain" AND random* OR PT randomized controlled trial OR PT clinical trial OR MH clinical trials+ OR AB groups OR OR placebo* OR doubl* N1 blind* OR singl* N1 blind* OR assign* OR AB allocat* OR volunteer*
Cochrane Central Register of Controlled Trials (EBSCO)	(Joint OR spinal OR spine OR thoracic OR cervical OR orthopedic OR orthopaedic OR thrust) N4 (manipulation* OR mobilization* OR mobilisation* OR manipulative) OR "manual therapy" OR "manual therapies" OR “manipulation therapies” OR “manipulation therapy” OR "manipulative therapy" OR "manipulative therapies" OR “mobilization therapy” OR “mobilization therapies” OR “mobilisation therapy” OR “mobilisation therapies” OR MH manipulation, spinal OR MH manipulation, orthopedic AND MH neck pain OR “neck pain” OR “cervical spine pain”
Pedro	“neck pain” manipulation*, “neck pain mobilisation*, “neck pain” mobilization*, “neck pain” “manual therapy”, “neck pain” “manual therapies”, “neck pain” “manipulative therapy”, “neck pain” “manipulative therapies”
AMED (OVID)	(mechanical neck pain OR neck pain) AND (thoracic manipulation OR thoracic thrust manipulation), mechanical neck pain AND thoracic manipulation, neck pain AND thoracic manipulation, neck pain AND manual therapy, neck pain AND manipulation
Clinicaltrials.gov	Conditioned searched: mechanical neck pain Other terms: thoracic manipulation

### Interventions

The intervention of interest in this systematic review was TSM. Manipulation has been defined as a high-velocity, small amplitude (grade V) therapeutic movement delivered at end-range [37]. Conversely, mobilization has been defined as a skilled therapeutic movement of varying speeds and amplitudes (grades I-IV) that does not include a high-velocity, small amplitude thrust [37]. For this systematic review several other interventions were compared to TS, which included placebo TSM, cervical spine manipulation, modalities, exercises, and standard care. Exercise provided in the studies included some combination of active range of motion, stretching, cervical and periscapular strengthening, and deep neck flexor endurance training [13, 17, 20, 22]. Standard care was operationally defined as the combination of exercise and/or modalities for the treatment of MNP [5, 13, 15, 17, 20, 38].

### Outcomes

The primary outcomes for this systematic review were pain and disability, with self-perceived rating of change and adverse events as secondary outcomes. Across all studies, pain was measured using either the Numeric Pain Rating Scale (NPRS; 0-10pts) or the Visual Analog Scale (VAS; 0-100mm). Disability was assessed using either the Neck Disability Index (NDI; 0–100%) or the Northwick Park Pain Questionnaire (NPQ; 0–100%). Previous research has identified the MCID values of the NPRS and VAS as 1.3 points (13 on a 100 point scale) [39] and 12 millimeters [40] respectively. Similarly, the MCID for the NDI and NPQ are 19% [39] and 25% [41] respectively. Self-perceived rating of change was measured using the Global Rating of Change (GROC). For the purposes of this review, and consistent with recent literature on neck pain, disability was operationally defined to include body structure and function impairments, participation restrictions, as well as personal and environmental factors that limit one’s ability to perform tasks on a daily basis [42, 43]. Adverse events and unwanted side effects were identified in several included studies through verbal communication between researcher and subjects.

Since the included studies collected data on outcome variables at different time points, the authors grouped these time points into immediate, short-term, and long-term follow-up periods. Immediate follow-up was defined as less than one week following interventions. Short-term follow-up was defined as any duration greater than one week and less three months, while long-term follow-up was defined as three months or greater. These time points were determined relative to the completion of TSM. In order to provide an overall assessment of the risk involved with TSM, included adverse events and unwanted side effects reported in the included studies were recorded. For this paper, an adverse event included any event following TSM that was not transient in nature and resulted in moderate to severe symptoms that required further treatment and was deemed unacceptable by the patient [19, 32, 44]. Unwanted side effects were defined as transient and did not require further treatment, including, but not limited to headache, soreness, fatigue, and increased pain [19, 32, 44].

### Methodological quality

The published criteria outlined by the Cochrane Collaboration was used to summarize the risk of bias for the included studies [35]. These criteria assessed sources of bias related to selection, performance, detection, attrition, and reporting. For each item, a plus sign was awarded and the article received a score of one if the criteria was fulfilled, while a minus sign or question mark was assigned if the criteria was not fulfilled or if it was unclear, respectively, and the article received a score of zero [35]. The sum of the awarded points represent the total risk of bias score out of a possible 12 points, with higher scores indicating a lower risk of bias in the included studies. Two authors (MM and KK) independently scored each of the included articles, with discrepancies resolved through further discussion until consensus was met (Table 2).

**Table 2:** Risk of bias criteria outline by the Cochrane Collaboration.

Study	Random Sequence Generation	Allocation Concealment	Blinding of Participants	Blinding of Providers	Blinding of Outcome Assessors	Incomplete Outcome Data–Drop Outs	Incomplete Outcome Data–ITT Analysis	Selective Reporting	Group Similarity at Baseline	Influence of Co-interventions	Compliance with Interventions	Timing of Outcomes Assessments	Total
Cleland et al 2005 [3]	+	+	+	-	+	+	+	+	+	+	+	+	11/12
Cleland et al 2007 [31]	+	+	-	-	+	+	+	+	+	+	+	+	10/12
Cleland et al 2010 [13]	+	+	-	-	+	+	+	+	?	+	+	+	9/12
Gonzalez-Iglesias et al 2009 [5]	+	+	+	-	+	+	+	+	+	+	+	+	11/12
Gonzalez-Iglesias et al 2009 [15]	+	+	+	-	+	+	+	+	+	+	+	+	11/12
Khoja et al 2015 [20]	+	+	-	-	+	-	-	+	+	-	+	+	7/12
Lau et al 2011 [17]	+	+	-	-	+	+	+	+	+	+	-	+	9/12
Lee et al 2016 [22]	+	+	-	-	+	+	-	+	+	-	?	+	7/12
Martinez-Segura et al 2012 [6]	+	+	-	-	+	+	+	+	+	+	+	+	10/12
Masaracchio et al 2013 [7]	+	+	-	-	+	+	+	+	+	+	+	+	10/12
Puentedura et al 2011 [32]	+	+	-	-	+	+	+	+	+	+	+	+	10/12
Salom-Moreno et al 2014 [29]	+	+	-	-	+	+	+	+	+	+	+	+	10/12
Sillevis et al 2010 [47]	+	+	+	-	+	+	+	+	+	+	+	+	11/12
Suvarnnato et al 2013 [30]	+	+	-	-	+	+	+	+	+	+	+	+	10/12

The risk of bias criteria outlined by the Cochrane Collaboration were used and results were labeled as + = criteria fulfilled;— = criteria not fulfilled; ? = unclear [35].

To provide an overall assessment of the quality of evidence, the authors used the GRADE approach. The GRADE approach consists of five domains, including risk of bias, inconsistency of results, indirectness of evidence, imprecision, and publication bias. Publication bias was assessed using combined methods: (1) a search of clinicaltrials.gov for grey literature that may not have been published due to non-significant results; (2) generation of funnel plots for each conducted meta-analysis, which plot the data around the overall estimated effect to assess for symmetry. Following evidence appraisal, outcomes are classified by level of evidence: [35, 45, 46].

- High quality evidence: further research is very unlikely to change our confidence in the estimate of effect, all domains are met.
- Moderate quality evidence: further research is likely to have an important impact on our confidence in the estimate of effect and may change the estimate, one of the domains is not met.
- Low quality evidence: further research is very likely to have an important impact on our confidence in the estimate of effect and is likely to change the estimate, two of the domains are not met.
- Very low quality evidence: any estimate of effect is very uncertain, three of the domains are not met.
- No evidence: no RCTs were identified that addressed this outcome.

### Data collection

Data extraction was performed by one author (KK), and all authors were consulted with any inquiries during the process. Extracted data included study details, participant demographics, interventions, follow-up time points, outcome measures, and a summary of results. Study authors were contacted in the event of missing data. For the purposes of this manuscript, participants who received TSM were labeled the TSM group, whereas participants who received other interventions were considered the comparison/control group and labeled as described in the individual studies (Table 3).

**Table 3:** Description of studies.

Study	Participant Characteristics	TSM Group	Comparison Group	Follow-up Time Points	Outcome Measures	Summary of Results
Cleland et al 2005 [3]	n = 36	Manipulation Supine TSM. A maximum of two manipulations were delivered per segment if no cavitation was heard on the first attempt. 1 treatment session	Placebo manipulation Supine TSM set-up position only. 1 treatment session	Immediately following intervention	VAS	TSM group demonstrated significant improvement in pain compared to the placebo group
	27 F, 9 M					
	Age^†^: 36y ± 9.8y					
	Symptom duration^†^:					
	TSM 12.2wks ± 3.5wks					
	CnG 13.2wks ± 4.2wks					
Cleland et al 2007 [31]	n = 60	Thoracic manipulation Supine TSM to upper thoracic spine* Supine TSM to middle thoracic spine* General cervical AROM exercises 1 treatment session	Thoracic mobilization One 30-second bout of grade III or IV central PA mobilization to the SP of T1-T6 General cervical AROM exercise 1 treatment session	2–4 days following intervention	NPRS NDI GROC	TSM group experienced significant improvements in disability and pain compared to the thoracic mobilization group TSM group exhibited significantly higher GROC scores at follow-up compared to the thoracic mobilization group
	33 F, 27 M					
	Age: 43.3y ± 12.7y					
	Symptom duration:					
	TSM 54.9d ± 46d					
	CG 56.1d ± 27.6d					
Cleland et al 2010 [13]	n = 140	Manipulation + exercise Visits 1 and 2 in the first week: Sitting distraction TSM to middle thoracic spine Supine TSM to upper thoracic spine Supine TSM to middle thoracic spine General cervical AROM exercises Visits 3–5 once weekly for the next 3 weeks: Stretching and strengthening program as described for the exercise-only group	Exercise-only Manual stretching by PT: UT, scalenes, SCM, levator scapulae, pectoralis major. Each stretch was held for 30 seconds x 2 Strengthening exercises: DNF endurance, cervical isometrics, and MT, LT, and SA strengthening. Each exercise was performed for 10 repetitions x 10 seconds 2 sessions in the first week and 1 session weekly for the next three weeks HEP x 1 daily	1 week of intervention (3^rd^ visit) 4 weeks of intervention (5^th^ visit) 6 months	NPRS NDI GROC	TSM group experienced significant improvements in disability and pain at each time point compared to the exercise-only group
	97 F, 43 M					
	Age: 39.9y ± 11.3y					
	Symptom duration:					
	TSM 62.5d ± 53.3d					
	CG 64.4d ± 61.3d					
Gonzalez-Iglesias et al 2009 [5]	n = 45	Manipulation + electrotherapy / thermal program Sitting distraction TSM.* TSM was performed once per week for 3 weeks Electrotherapy / thermal modalities were applied at 2 sessions per week for 3 weeks	Electrotherapy / thermal program Infrared lamp, 50 cm distance from patient’s neck for 15 minutes TENS, electrodes placed bilaterally to spinous process of C7 for 20 minutes 2 sessions per week for 3 weeks	1 week after DC	NPRS NPQ	TSM group experienced significant improvements in disability and pain compared to the electrotherapy / thermal program group
	25 F, 20 M					
	Age: 34y ± 4y					
	Symptom duration:					
	TSM 18d ± 6d					
	CG 17d ± 5d					
Gonzalez-Iglesias et al 2009 [15]	n = 45	Manipulation + electro / thermal program Sitting distraction TSM* TSM was performed once per week for 3 weeks Electrotherapy / thermal modalities were applied at 2 sessions per week for 3 weeks	Electro / thermal therapy program Infrared lamp, 50 cm distance from patient’s neck for 15 minutes TENS, electrodes placed bilaterally to spinous process of C7 for 20 minutes. 2 sessions per week for 3 weeks	3 weeks of intervention (5^th^ visit) 2 weeks after DC 4 weeks after DC	VAS NPQ	TSM group experienced significant improvement in pain at each time point compared to the electro / thermal therapy program group TSM group experienced significant improvement in disability at DC and 2-week follow-up compared to the electro / thermal therapy program group
	21 F, 24 M					
	Age: 34y ± 5y					
	Symptom duration:					
	TSM 19.5d ± 4.5d					
	CG 18.7d ± 3.9d					
Khoja et al 2015 [20]	n = 22	Multimodal neck program + thoracic manipulation Standing or sitting TSM. TSM and multimodal neck program was performed at each treatment session. 2 sessions per week for 6 weeks HEP: Wing arm exercise each day to maintain thoracic ROM	Multimodal neck program Electro / thermal therapeutic agents: TENS, heat, or US. Active exercises: neck ROM, chin tucks, isometric strengthening exercises for neck flexion, extension, and lateral bending Manual therapy: STM to the neck and shoulder girdle and oscillatory lateral glides to the cervical spine Each of the 3 interventions were used pragmatically as decided by the PT for 7–15 minutes 2 sessions per week for 6 weeks	2 weeks of intervention 4 weeks of intervention 6 weeks of intervention Subjects were DC as they recovered. Therefore, not all subjects underwent 6 weeks of intervention	NPRS NDI GROC	No significant between group differences found for any outcome measure
	15 F, 7 M					
	Age: 38y ± 11y					
	Symptom duration:					
	TSM <3mo n = 7, 3-6mo n = 2, >6mo n = 2					
	CG <3mo n = 6, 3-6mo n = 2, >6mo n = 2					
Lau et al 2011 [17]	n = 120	Thoracic manipulation Supine TSM. Infrared radiation therapy for 15 minutes over the painful site Education materials about pathology of neck pain and exercises: neck AROM, neck isometrics, and stretching of UT and scalenes 2 sessions per week for 4 weeks	Control Infrared radiation therapy for 15 minutes over the painful site Education materials about pathology of neck pain and exercises: neck AROM, neck isometrics, and stretching of UT and scalenes. 2 sessions per week for 4 weeks	4 weeks of intervention (8^th^ visit) 3 months 6 months	NPRS NPQ	TSM group experienced significant improvements in pain and disability at each time point compared to the control group
	60 F, 60 M					
	Age: 43.78y ± 9.25y					
	Symptom duration: N/A					
Lee et al 2016 [22]	n = 46	Group A: Thoracic manipulation + DNF training Supine TSM. DNF training Craniocervical flexion with biofeedback Self-stretch: UT and levator scapulae 35 minutes per session, 3 sessions per week for 10 weeks	Group B: DNF training DNF training. Craniocervical flexion with biofeedback 35 minutes per session, 3 sessions per week for 10 weeks Group C: Control Cervical AROM exercises 35 minutes per session, 3 sessions per week for 10 weeks	10 weeks of intervention (30^th^ visit)	VAS Korean NDI	TSM group experienced significant improvements in pain and disability after 10 weeks of treatment compared to DNF training and control groups
	Sex: N/A					
	Age: N/A					
	Symptom duration:					
	TSM 20.7mo					
	CG 19.4mo					
	CnG 11.8mo					
Martinez-Segura et al 2012 [6]	n = 90	Thoracic manipulation Supine TSM to the upper thoracic spine*	Right cervical manipulation Supine cervical manipulation on the right side* Left cervical manipulation Supine cervical manipulation on the left side*	10 minutes following intervention	NPRS	No significant between group differences for pain
	46 F, 44 M					
	Age: 37y ± 8y					
	Symptom duration:					
	TSM 3.8y ± 1.5y					
	CG(R) 3.7y ± 1.5y					
	CG(L) 3.5y ± 1.4y					
Masaracchio et al 2013 [7]	n = 66	Thoracic manipulation + cervical mobilization + HEP Supine TSM to upper thoracic spine x 2. Supine TSM to middle thoracic spine x 2. Cervical spine mobilization. One bout of 10 PA oscillations grade III mobilization to each spinous processes of C3-C7. HEP: Cervical AROM exercise 2 sessions, 2–3 days apart	Cervical mobilization + HEP Cervical spine mobilization. One bout of 10 PA oscillations grade III mobilization to each spinous processes of C3-C7 HEP: Cervical AROM exercise 2 sessions, 2–3 days apart	1 week following intervention	NPRS NDI GROC	TSM group experienced significant improvements in pain and disability compared to the mobilization + HEP only group TSM group exhibited significantly higher GROC scores at follow-up compared to the mobilization + HEP only group
	50 F, 16 M					
	Age: 32.5y ± 11.4y					
	Symptom duration:					
	TSM 37.3d ± 25.3d					
	CG 34.5d ± 26.9d					
Puentedura et al 2011 [32]	n = 24	Thoracic manipulation + exercise program Visits 1 and 2: Sitting distraction TSM to middle thoracic spine* Supine TSM to upper thoracic spine* Supine TSM to middle thoracic spine* 3-finger exercise for cervical rotation Visits 3–5: 3-finger exercise for cervical rotation Bilateral shoulder shrugs and scapular retraction Bilateral shoulder horizontal abduction and adduction Upper cervical flexion and extension Lower cervical flexion and extension. Theraband rows Lateral pull downs. Visits 1–3 took place in the first week and visits 4–5 took place in the second week of therapy	Cervical manipulation + exercise program Visits 1 and 2: Supine cervical thrust manipulation to both sides of the neck* 3-finger exercise for cervical rotation Visits 3–5: 3-finger exercise for cervical rotation Bilateral shoulder shrugs and scapular retraction Bilateral shoulder horizontal abduction and adduction Upper cervical flexion and extension Lower cervical flexion and extension Theraband rows Lateral pull downs Visits 1–3 took place in the first week and visits 4–5 took place in the second week of therapy	1 week of intervention (4^th^ visit) 4 weeks 6 months	NPRS NDI GROC	No significant between group differences in disability at 1 week and 4-week follow-up, however, the cervical manipulation group experienced significant improvement in disability compared to the TSM group at 6-month follow-up The cervical manipulation group experienced significant reduction in pain at each time point compared to the TSM group TSM group exhibited significantly higher GROC scores at 1 week compared to the cervical manipulation group Cervical manipulation group exhibited significantly higher GROC scores at 4-week and 6-month follow-up compared to the TSM group
	16 F, 8 M					
	Age: 33.7y ± 6.4y					
	Symptom duration:					
	TSM 18.8d ± 9.3d					
	CG 11.5d ± 7.0d					
Salom-Moreno et al 2014 [29]	n = 52	Thoracic manipulation Supine TSM to the middle thoracic spine*	Thoracic mobilization 2 minutes of 20-second bouts of grade III-IV central PA mobilization to the SP of T3-T6	10 minutes after intervention	NPRS	TSM group experienced significant improvement in pain compared to the thoracic mobilization group
	22 F, 30 M					
	Age: 33y ± 9y					
	Symptom duration:					
	TSM 2.2y ± 1.1y					
	CG 2.4y ± 1.3y					
Sillevis et al 2010 [47]	n = 100	Manipulation Supine TSM to the upper thoracic spine T3-T4	Placebo Clinician placed flat hand under T4 segment with patient in supine	Immediately following intervention	VAS	No significant between group differences in pain scores
	77 F, 23 M					
	Age: TSM 42.7y					
	Placebo 46.84y					
	Symptom duration:					
	TSM 23.3mo					
	CG 25.3mo					
Suvarnnato et al 2013 [30]	n = 39	Thoracic manipulation Prone TSM to the middle thoracic spine T6-T7* 2-minute session	Thoracic mobilization 1 minute of unilateral grade III PA mobilization to left and right facet joint of T6-T7 in prone 2-minute session Control Prone lying with PT’s hands resting on the facet joint at T6-T7 2-minute session	Immediately following intervention 24 hours	VAS	No significant between group differences in pain immediately post intervention or at 24-hour follow-up
	29 F, 10 M					
	Age: TSM 37y ±12.49y					
	Symptom duration: inclusion criteria indicates >3mo, no demographic data provided					

Abbreviations: AROM, active range of motion; CG. Comparison group; CnG, control group; DNF, deep neck flexor; DC, discharge; FPS, Faces Pain Scale; F, females; GROC, Global Rating of Change; HEP, home exercise program; HVLA, high-velocity low-amplitude; LT, lower trapezius; M, males; MT, middle trapezius; N/A, data not available; NDI, Neck Disability Index; NPQ, Northwick Park Neck Pain Questionnaire; NPRS, Numerical Pain Rating Scale; PT, physical therapist; PA, posterior to anterior; ROM, range of motion; SA, serratus anterior; STM, soft tissue mobilization; SP: spinous process; SCM, sternocleidomastoid; TSM: thoracic spine manipulation; TENS, transcutaneous electrical stimulation; US, ultrasound; UT, upper trapezius; VAS, visual analog scale.

*A maximum of two manipulations were delivered if no cavitation was heard on the first attempt.

^†^All values for age symptoms duration are reported as mean ± standard deviation when data is available

### Data analysis

Data analysis was conducted using Revman 5.3. When available, change scores and standard deviations (SD) were inputted into the meta-analysis. In the absence of change scores, post-test measures were alternately used. When included studies provided 95% confidence intervals (CI) rather than SDs, the authors calculated SDs for the purposes of meta-analysis [48]. For all meta-analyses, a random-effects model with inverse variance methods was used to calculate mean differences and 95% CI. When multiple data points existed in individual studies within an operationally defined time point, data from the longest follow-up time period was used for meta-analysis purposes (Table 4). Mean differences were calculated so that minimal clinical important differences (MCID) of the primary outcome measures could be discussed in relation to patient improvement. Therefore, for pain and disability outcomes, scores were converted to a common (0–100) point scale. This would provide a more comprehensive understanding of the data, providing both statistical and clinical significance. Statistical heterogeneity was evaluated using the I^2^ statistic, with values of more than 50% indicating considerable levels of heterogeneity [49]. Where statistical pooling was not possible, either due to missing data or heterogeneity between studies, findings were presented in narrative form.

**Table 4:** Results of included studies.

Study	Outcome	TSM group^§^		Comparison group^§^		Between group differences	
						(95% CI)	
Cleland et al 2005 [3]	VAS**	Pre	41.6 ± 17.8	Pre	47.7 ± 18.4		
		Post	26.1 ± 17.2	Post	43.5 ± 19.5		
		Δ score	15.5 ± 7.7	Δ score	4.2 ± 4.6	Immediate^^^	^‡^
Cleland et al 2007 [31]	NPRS**	Pre	5.3 ± 1.4	Pre	4.5 ± 2.1		
		2–4 d	2.7 ± 1.4	2–4 d	3.9 ± 2.2		
		Δ score	2.6 ± 1.5	Δ score	0.54 ± 1.07	2–4 d	2.03% (1.4, 2.7)^‡^
	NDI**	Pre	33.5 ± 11.2	Pre	29.6 ± 12.6		
		2–4 d	18.0 ± 10.9	2–4 d	24.0 ± 13.4		
		Δ score	15.5 ± 9.3	Δ score	5.5 ± 8.8	2–4 d	10.03% (5.3, 4.7)^‡^
	GROC					2–4 d	1.5 points higher in TSM group (0.48, 2.5)
Cleland et al 2010 [13]	NPRS	Pre	4.4 ± 1.5	Pre	3.9 ± 1.4		
		1 wk	2.3 ± 0.90	1 wk	3.0 ± 1.4	1 wk	-0.70 (-1.1, -0.32)^‡^
		4 wk	1.7 ± 0.91	4 wk	1.9 ± 1.0	4 wk	-0.19 (-0.53, 0.16)
		6 mo	1.4 ± 0.89	6 mo	1.8 ± 1.0	6 mo	-0.35 (-0.75, 0.04)
		Δ score	NA	Δ score	NA		
	NDI	Pre	29.5 ± 8.2	Pre	28.6 ± 7.2		
		1 wk	14.8 ± 6.3	1 wk	18.4 ± 8.2	1 wk	-3.6 (-6.0, -1.2)^‡^
		4 wk	10.1 ± 5.6	4 wk	13.5 ± 6.5	4 wk	-3.5 (-5.6, -1.3)^‡^
		6 mo	7.1 ± 3.7	6 mo	11.7 ± 7.2	6 mo	-4.6 (-7.0, -2.2)^‡^
		Δ score	NA	Δ score	NA		
	GROC	1 wk	18.5% of participants +5 or greater	1 wk	11.4% of participants +5 or greater	1 wk	NS
		4 wk	51.4% of participants +5 or greater	4 wk	31.4% of participants +5 or greater	4 wk	^†^
		6 mo	80% of participants +5 or greater	6 mo	35.7% of participants +5 or greater	6 mo	*
Gonzalez-Iglesias et al 2009 [5]	NPRS	Pre	5.6 ± 0.9	Pre	5.37 ± 0.6		
		1 wk	2.3 ± 1.0	1 wk	4.3 ± 0.8		
		Δ score	32.8 ± 7.24	Δ score	9.4 ± 5.02	1 wk	2.3 (2.0, 2.7)^‡^
	NPQ**	Pre	27.8 ± 3.1	Pre	27.1 ± 2.7		
		1 wk	15.2 ± 4.1	1 wk	22.9 ± 2.9		
		Δ score	12.6 ± 2.93	Δ score	4.1 ± 1.69	1 wk	8.5 (7.2, 9.8)^‡^
Gonzalez-Iglesias et al 2009 [15]	VAS	Pre	54.7 ± 8.2	Pre	52.7 ± 5.5		
		DC	20.2 ± 7.8	DC	44.7 ± 5.5	DC	26.5 (22.9, 30.2)^‡^
		2 wk	26.4 ± 11.8	2 wk	41.2 ± 6.1	2 wk	16.8 (11.7, 21.8)^‡^
		4 wk	21.5 ± 10.6	4 wk	42.2 ± 7.7		
		Δ score	33.2 ± 9.06	Δ score	10.4 ± 8.72	4 wk	22.8 (17.7, 27.8)^‡^
	NPQ	Pre	27.9 ± 3.0	Pre	27.0 ± 3.1		
		DC	15.2 ± 3.9	DC	23.1 ± 3.2	DC	8.8 (7.5, 10.1)^‡^
		2 wk	14.7 ± 2.8	2 wk	21.8 ± 3.3		
		Δ score	13.2 ± 4.17	Δ score	5.1 ± 3.71	2 wk	8.0 (5.8, 10.2)^‡^
Khoja et al 2015 [20]	NPRS	Pre	5.0 ± 1.7	Pre	5.7 ± 1.4		
		6 wk	2.1 ± 2.4	6 wk	2.9 ± 2.3		
		Δ score	2.9 ± 2.09	Δ score	2.7 ± 2.38	6 wk	0.2 (-1.7, 2.1)
	NDI	Pre	32.2 ± 9.4	Pre	33.0 ± 12.3		
		6 wk	17.6 ± 15.2	6 wk	21.3 ± 18.7		
		Δ score	14.6 ± 15.18	Δ score	11.8 ± 22.25	6 wk	2.9 (-11.5, 17.2)
	GROC	6 wk	70% of participants moderately better (+4)	6 wk	50% of participants moderately better (+4)		
Lau et al 2011 [17]	NPRS	Pre	5.02 ± 1.83	Pre	5.05 ± 1.48		
		DC	3.14 ± 1.99	DC	4.37 ± 1.75	DC	(3.33, 4.05)^†^
		3 mo	3.29 ± 1.70	3 mo	4.41 ± 2.02	3 mo	(3.46, 4.19)^†^
		6 mo	2.98 ± 1.76	6 mo	4.24 ± 2.12	6 mo	(3.23, 3.99)^†^
		Δ score	NA	Δ score	NA		
	NPQ	Pre	39.15 ± 15.00	Pre	41.86 ± 11.66		
		DC	27.15 ± 16.84	DC	36.01 ± 13.47	DC	(28.58, 34.52)*
		3 mo	27.84 ± 15.8	3 mo	35.40 ± 14.4	3 mo	(28.49, 34.40)*
		6 mo	28.77 ± 16.03	6 mo	34.80 ± 15.34	6 mo	(28.71, 34.86)*
		Δ score	NA	Δ score	NA		
Lee et al 2016 [22]	VAS	Pre	52 ± 6	Pre (DNF)	51 ± 6		TSM greater improvement in pain at DC after 10 wk of treatment*
		DC	14 ± 5	DC (DNF)	25 ± 5		
		Δ score	38 ± 6	Δ score	26 ± 6	DC	
				Pre (C)	53 ± 6		
				DC (C)	38 ± 4		
				Δ score	15 ± 5		
	Korean NDI	Pre	27.6 ± 4.5	Pre (DNF)	27.2 ± 3.4		TSM greater improvement in disability at DC after 10 wk of treatment*
		DC	6.6 ± 2.1	DC (DNF)	10.7 ± 1.8		
		Δ score	21.0 ± 3.6	Δ score	16.5 ± 4.0	DC	
				Pre (C)	27.1 ± 3.9		
				DC (C)	20.4 ± 2.5		
				Δ score	6.7 ± 3.4		
Martinez-Segura et al 2012 [6]	NPRS	Pre	5.7 ± 1.2	Pre (R)	5.6 ± 1.7		
		Post	2.9 ± 1.6	Post (R)	2.9 ± 2.0		
		Δ score	2.8 ± 1.61	Δ score	2.7 ± 1.51	Immediate^^^	NS
				Pre (L)	5.6 ± 1.2		
				Post (L)	2.8 ± 1.7		
				Δ score	2.8 ± 1.64		
Masaracchio et al 2013 [7]	NPRS	Pre	5.1 ± 1.2	Pre	4.9 ± 1.7		
		1 wk	2.2 ± 0.9	1 wk	3.5 ± 1.6		
		Δ score	2.8 ± 6.61	Δ score	1.5 ± 6.9	1 wk	1.3 (0.7, 2.0)^‡^
	NDI	Pre	28.5 ± 8.6	Pre	26.3 ± 8.4		
		1 wk	12.3 ± 6.2	1 wk	18.9 ± 8.4		
		Δ score	16.2 ± 40.73	Δ score	7.4 ± 21.05	1 wk	8.8 (5.4, 12.2)^‡^
	GROC	1 wk	94% of participants moderately better (+4). Participants average moderately better (+4).	1 wk	35% of participants moderately better (+4). Participants average a little bit better (+2).	1 wk	2 points higher in TSM group (1, 3)^‡^
Puentedura et al 2011 [32]	NPRS	Pre	3.6 ± 1.4	Pre	4.6 ± 2.2		
		1 wk	2.1 ±1.6	1 wk	0.1 ± 0.2	1 wk	^†^
		4 wk	1.9 ± 1.0	4 wk	0.1 ± 0.1	4 wk	^‡^
		6 mo	2.3 ± 1.1	6 mo	0.1 ± 0.1	6 mo	^‡^
		Δ score	NA	Δ score	NA		
	NDI	Pre	12.6 ± 1.9	Pre	13.4 ± 2.9		
		1 wk	10.9 ± 2.0	1 wk	8.3 ± 3.4	1 wk	NS
		4 wk	9.1 ± 3.7	4 wk	4.2 ± 5.4	4 wk	NS
		6 mo	9.9 ± 3.9	6 mo	3.7 ± 5.7	6 mo	^†^
		Δ score	NA	Δ score	NA		
	GROC		20% of participants quite a bit better (+5). Participants average moderately better (+4) at 1 wk and 4 wk, somewhat better (+3) at 6 mo.		100% of participants quite a bit better (+5). Participants average a great deal better (+6) at 1 wk, a very great deal better (+7) at 4 wk and 6 mo.		Cervical manipulation group higher GROC scores at 1 wk, 4 wk, and 6 mo^‡^
Salom-Moreno et al 2014 [29]	NPRS	Pre	6.0 ± 1.4	Pre	5.8 ± 1.2		
		Post	2.5 ± 1.7	Post	3.7 ± 1.5		
		Δ score	3.5 ± 1.35	Δ score	2.1 ± 1.0	Immediate^^^	1.4 (0.8, 2.1)^‡^
Sillevis et al 2010 [47]	VAS	Pre	38	Pre	33		
		Post	32	Post	28		
		Δ score	5.3	Δ score	4.3	Immediate^^^	NS
Suvarnnato et al 2013 [30]	VAS	Pre	45.08 ± 18.87	Pre (C)	43.69 ± 15.60		
		Post	37.46 ± 19.57	Post (C)	38.00 ± 18.12	Immediate^^^	NS
		24 hr	35.92 ± 19.77	24 hr (C)	35.08 ± 14.41	24 hr	NS
		Δ score	NA	Δ score	NA		
				Pre (M)	46.62 ± 16.66		
				Post (M)	38.08 ± 16.66		
				24 hr (M)	35.15 ± 18.66		
				Δ score	NA		

Abbreviations: Δ score, change score (within group difference); C, control group; DNF, deep neck flexor training group; DC, discharge; FPS, Faces Pain Scale; F/U, follow-up; GROC, Global Rating of Change; L, left cervical manipulation group; M, mobilization group; MNP, multimodal neck program; NDI, Neck Disability Index; NPQ, Northwick Park Neck Pain Questionnaire; NA, not applicable; NS, not significant; NPRS, Numerical Pain Rating Scale; R, right cervical manipulation group; SD, standard deviation; TS, thoracic spine; TSM, thoracic spine manipulation; TX, treatment; VAS, Visual Analog Scale.

*P < 0.05

^†^P < 0.01

^‡^P < 0.001

^§^Values are reported as mean ± standard deviation unless otherwise noted.

^^^Immediate follow-up was defined as less than one week following interventions

** The NPRS and VAS range from 0–10 points and 0–100 millimeters, respectively, where lower scores represent less pain and higher scores represent greater pain. The NDI and NPQ both range from 0% to 100%, where lower scores represent less disability and higher scores represent greater disability.

Separate meta-analyses were performed comparing TSM to other interventions, including placebo thoracic manipulation, thoracic or cervical spine mobilization, standard care, and cervical spine manipulation for their effect on pain and disability. Within the TSM versus mobilization meta-analysis, studies were included that compared TSM to thoracic or cervical mobilization. A subsequent subgroup analysis was conducted to isolate a pooled effect size for the comparison of TSM to only thoracic mobilization in order to eliminate cervical mobilization as a confounding treatment variable. Separate meta-analyses for pain and disability comparing TSM to standard care were conducted for both short-term and long-term follow-up time periods.

Although the authors originally intended to perform further subgroup analysis based on chronicity of symptoms, this was not feasible as the majority of included studies did not adequately report on symptom duration. Furthermore, many studies included cohorts of patients that presented with a wide range of symptom duration, thus making it impossible to allocate patients into acute (< 6 weeks), subacute (6 to 12 weeks), or chronic (> 12 weeks) phases of symptom duration as recently suggested by the Cochrane Collaboration [35].

## Results

### Study selection

**Fig 1:**
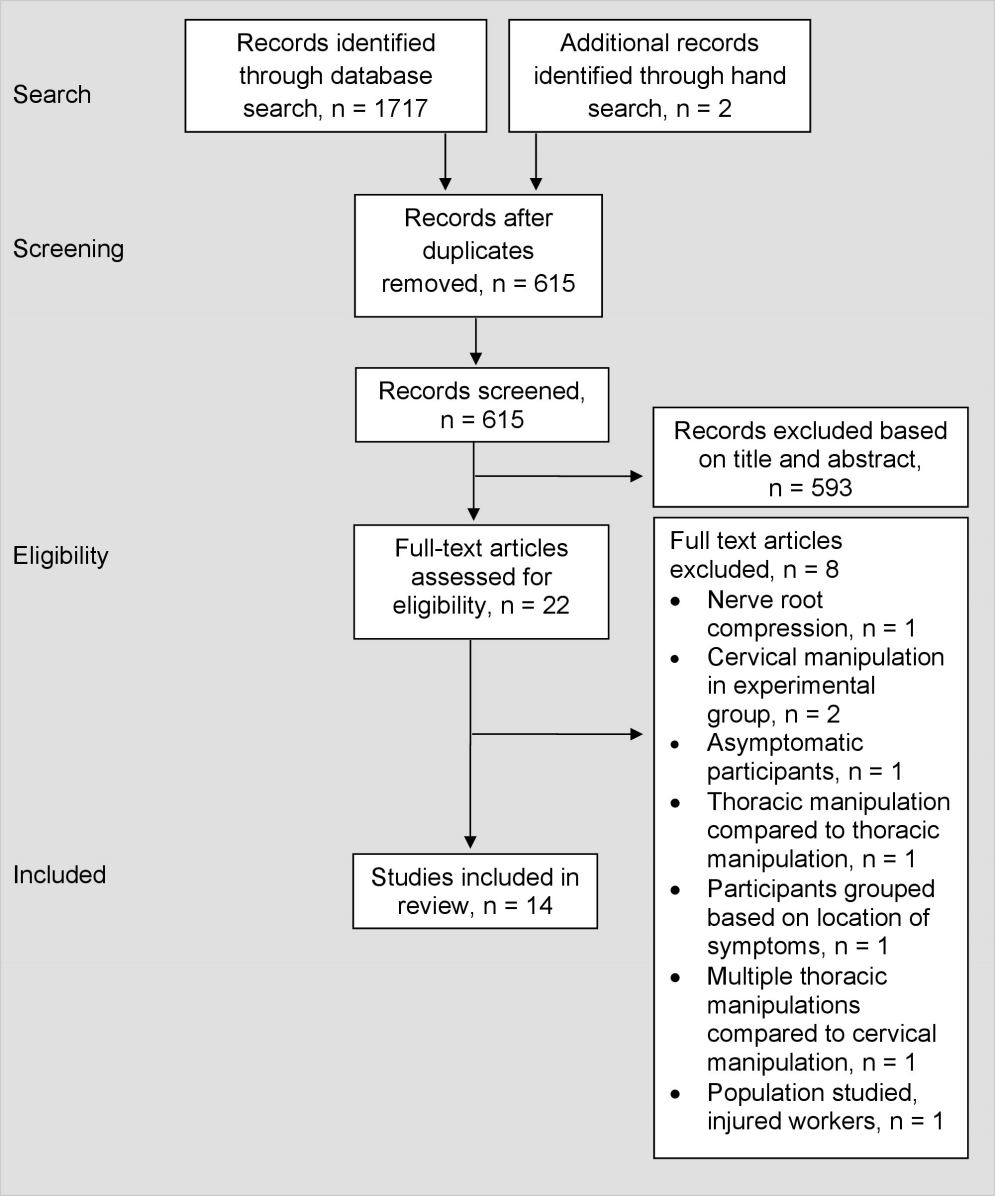
PRISMA flow diagram of search results and studies included.

The search strategy identified 1,717 studies, with two additional studies located through manual searching. Twenty-two full text articles were assessed to determine eligibility and 14 met the inclusion criteria (Fig 1). Thirteen [3, 5–7, 13, 15, 17, 20, 22, 29–32] of the 14 studies were included in various meta-analyses. One study, Sillevis et al [47], could not be included in the pooled analyses because of missing standard deviation values that were unattainable after contacting the author.

### Characteristics of included trials

The included 14 studies [3, 5–7, 13, 15, 17, 20, 22, 29–32, 47] were individually scored for their risk of bias (Table 2). The average score was 9.07 out of 12 (range 7–12), with a median score of 10 on the Cochrane Collaboration risk of bias criteria. Across the included studies, there was increased risk of bias for inadequate provider and participant blinding [35]. While participant blinding is possible in manual therapy trials, only four out of 14 studies provided adequate information on participant blinding. Blinding of the providers (clinicians) who administered the interventions is not possible in manual therapy trials. In addition, because all the included studies were comparing the effectiveness of interventions, it is also important that the groups started out similar at baseline. This was explicitly stated in 13 out of 14 included studies, but was unclear in one study, Cleland et al 2010 [13].

A total of 885 participants were included across 14 studies. Among studies that reported the distribution of male and female participants (13), 61.7% were female and 38.3% were male (Table 3) [3, 5–7, 13, 15, 17, 20, 29–32, 47]. All 14 studies included participants with MNP that were randomly assigned to either the TSM group or a comparison group (12 studies) [3, 5–7, 13, 15, 17, 20, 29, 31, 32, 47]. In two studies [22, 30], there was also a control group. In Lee et al [22], participants in the control group received cervical active range of motion exercises, while in Suvarnnato et al [30], participants in the control group received placebo thoracic mobilization.

All 14 studies [3, 5–7, 13, 15, 17, 20, 29–32, 38, 47] assessed pain (NPRS or VAS), nine studies [5, 7, 13, 15, 17, 20, 31, 32, 38] assessed disability (NDI or NPQ), five studies [7, 13, 20, 31, 32] assessed self-perceived rating of change (GROC), and nine studies reported on adverse events and unwanted side effects [3, 6, 7, 13, 17, 22, 29, 31, 32]. Both pain and disability measures [5, 7, 13, 15, 17, 20, 22, 31, 32] were reported in nine studies, while four studies [7, 13, 31, 32] reported on all four outcomes (pain, disability, self-perceived rating of change, and adverse events/unwanted side effects. Three studies [3, 30, 47] compared TSM to placebo TSM, two of which were included in the meta-analysis [3, 30]. Four studies compared TSM to mobilization, with three [29, 30, 50] comparing TSM to thoracic mobilization and one [7] comparing TSM to cervical mobilization. Six studies [5, 13, 15, 17, 20, 22] compared TSM to standard care. Two studies [6, 32] compared TSM to cervical spine manipulation (Table 3).

### Outcomes

Data from the included studies for the TSM group, the comparison and control groups, as well as between group differences, are presented in Table 4.

#### Pain

**Fig 2:**
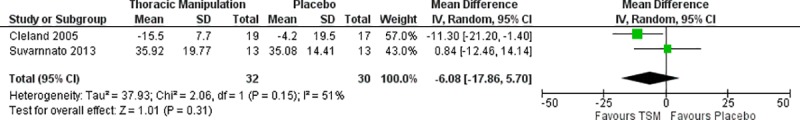
Meta-analysis of TSM versus placebo TSM for pain at immediate follow-up.

**Fig 3:**
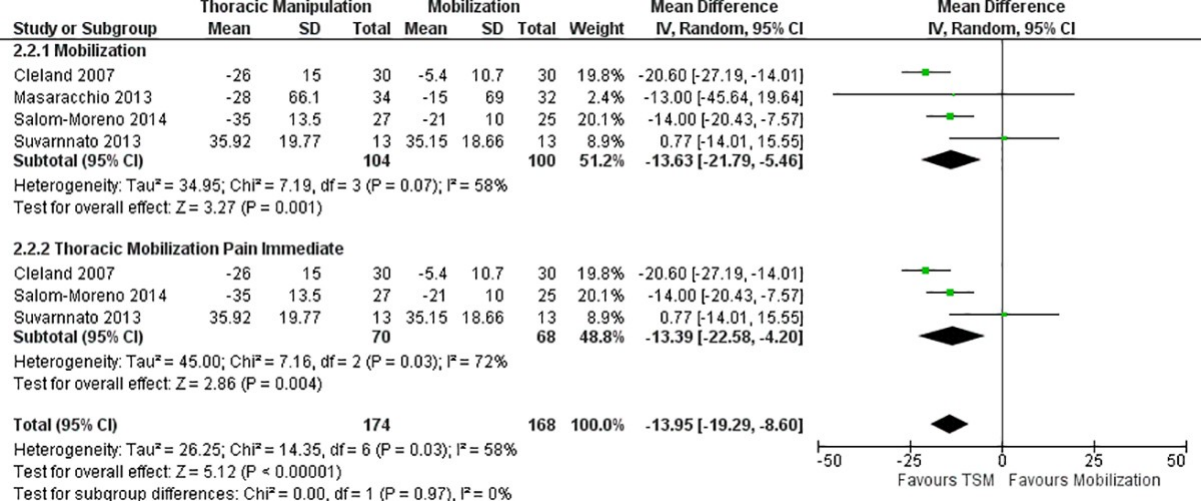
Meta-analysis of TSM versus mobilization for pain without a distinction between immediate and short-term follow-up.

**Fig 4:**
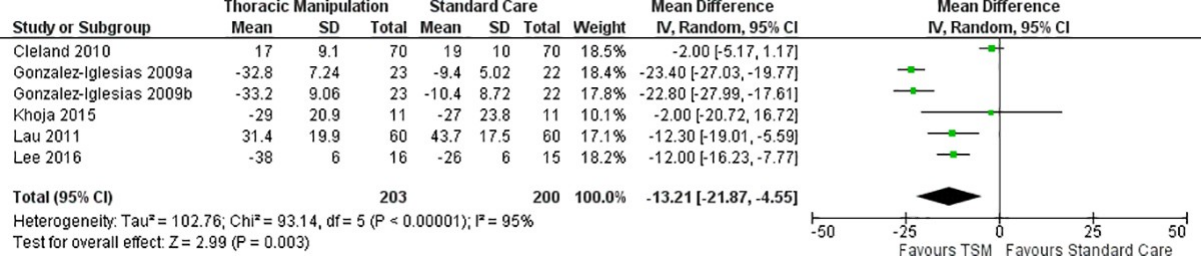
Meta-analysis of TSM versus standard care for pain at short-term follow-up.

**Fig 5:**
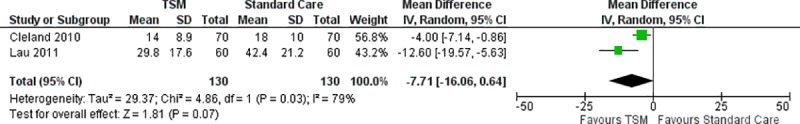
Meta-analysis of TSM versus standard care for pain at long-term follow-up.

**Fig 6:**
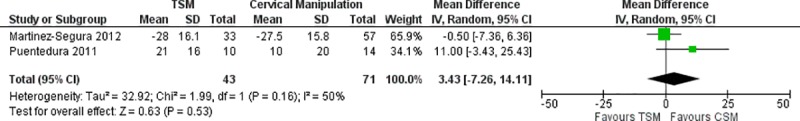
Meta-analysis of TSM versus cervical manipulation for pain without a distinction between immediate and short-term follow-up.

Pain was measured across all 14 studies using either the NPRS or VAS. Separate meta-analyses were conducted to compare TSM to various interventions. Meta-analysis of two studies [3, 30] (n = 62) that compared TSM to placebo TSM revealed a non-significant effect (MD -6.08; 95% CI: -17.86, 5.70; I^2^ = 51%, p = 0.31) at immediate follow-up (Fig 2). Meta-analysis of four studies [7, 29–31] (n = 204) that compared TSM to mobilization revealed a significant effect favoring the TSM group (MD -13.63; 95% CI: -21.79, -5.46; I^2^ = 58%, p = 0.001) without a distinction between immediate and short-term follow-up (Fig 3). A subgroup analysis of three studies [29–31] (n = 138) that compared TSM to only thoracic mobilization yielded similar results with a significant effect favoring the TSM group (MD -13.39; 95% CI: -22.58, -4.20; I^2^ = 72%, p = 0.004) at immediate follow-up (Fig 3). Meta-analysis of six studies [5, 13, 15, 17, 20, 22] (n = 403) that compared TSM to standard care revealed a significant effect favoring the TSM group (MD -13.21; 95% CI: -21.87, -4.55; I^2^ = 95%, p = 0.003) at short-term follow-up (Fig 4), while two studies [13, 17] (n = 260) revealed a non-significant effect favoring the TSM group (MD -7.71; 95% CI: -16.06, 0.64; I^2^ = 79%, p = 0.07) at long-term follow-up (Fig 5). Meta-analysis of two studies [6, 32] (n = 114) that compared TSM to cervical spine manipulation revealed a non-significant effect (MD 3.43; 95% CI: -7.26, 14.11; I^2^ = 50%, p = 0.53) without a distinction between immediate and short-term follow-up (Fig 6).

#### Disability

**Fig 7:**
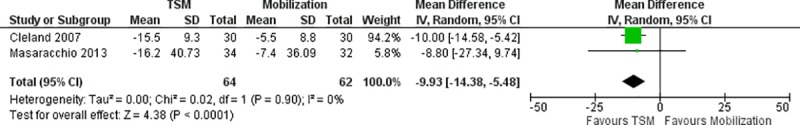
Meta-analysis for TSM versus mobilization for disability without a distinction between immediate and short-term follow-up.

**Fig 8:**
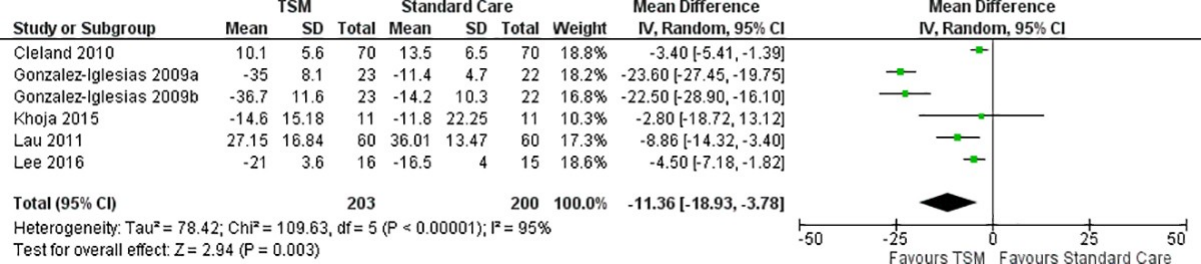
Meta-analysis for TSM versus standard care for disability at short-term follow-up.

**Fig 9:**
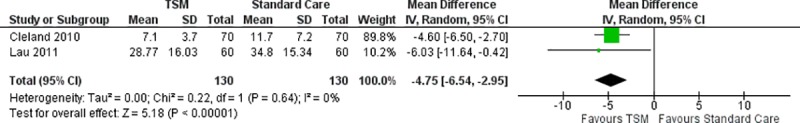
Meta-analysis for TSM versus standard care for disability at long-term follow-up.

Disability was measured across nine studies [5, 7, 13, 15, 17, 20, 22, 31, 32] using either the NDI or the NPQ. Meta-analysis of two studies [7, 31] (n = 126) that compared TSM to mobilization revealed a significant effect favoring the TSM group (MD -9.93; 95% CI: -14.38, -5.48; I^2^ = 0%, p < 0.00001) without a distinction between immediate and short-term follow-up (Fig 7). Meta-analysis of six studies [5, 13, 15, 17, 20, 22] (n = 403) that compared TSM to standard care revealed a significant effect favoring the TSM group (MD -11.36; 95% CI: -18.93, -3.78; I^2^ = 95%, p = 0.003) at short-term follow-up (Fig 8), while two studies [13, 17] (n = 260) revealed a significant effect favoring the TSM group (MD -4.75; 95% CI: -6.54, -2.95; I^2^ = 0%, p < 0.00001) at long-term follow-up (Fig 9).

#### Global rating of change

While it has been common in recent years to assess self-perceived level of function following interventions, only five [7, 13, 20, 31, 32, 47] of the 14 studies included in this systematic review reported on the GROC. Four of those studies demonstrated higher scores on the GROC in the group that received TSM [7, 13, 20, 31, 47], while one [32] favored cervical manipulation compared to TSM.

#### Adverse events and unwanted side effects

Nine of the included studies [3, 6, 7, 13, 17, 22, 29, 31, 32] reported on adverse events and unwanted side effects associated with both the TSM and comparative treatment interventions. A qualitative summary of the data, including number of subjects who experienced symptoms and the duration of symptoms, is presented in Table 5. None of the subjects in any of these nine studies reported any adverse events, while only four of the studies [6, 29, 31, 32] reported unwanted side effects. Of these four studies, two [6, 31] found that there was no significant difference between the TSM group and the comparison group, one [29] did not perform between group differences, and one [32] found greater unwanted side effects in the TSM group than the cervical manipulation group. In three of the studies [6, 31, 32], the duration of unwanted side effects lasted less than 24 hours, while in one study [29], they lasted less than 12 hours.

**Table 5:** Adverse events* and un-wanted side effects^†^ reported in included studies.

Study	Un-wanted side effects		Between group differences
	TSM group	Comparison group	
Cleland et al 2005 [3]	None reported^‡^	None reported	None reported
Cleland et al 2007 [31]	n = 10	n = 9	Not significant
	Aggravation of symptoms (8)	Aggravation of symptoms (2)	
	Muscle spasm (1)	Muscle spasm (1)	Duration of symptoms <24
	Headache (1)	Neck stiffness (2)	hours.
		Headache (2)	
		Radiating symptoms (2)	
Cleland et al 2010 [13]	None reported	None reported	None reported
Gonzalez-Iglesias et al 2009 [5]	None mentioned^§^	None mentioned	None mentioned
Gonzalez-Iglesias et al 2009 [15]	None mentioned	None mentioned	None mentioned
Khoja et al 2015 [20]	None mentioned	None mentioned	None mentioned
Lau et al 2011 [17]	None reported	None reported	None reported
Lee et al 2016 [22]	None reported	None reported	None reported
Martinez-Segura et al 2012 [6]	n = 1	n = 1	Not significant
	Neck fatigue	Increased neck pain	
			Duration of symptoms <24
			hours.
Masaracchio et al 2013 [7]	None reported	None reported	None reported
Puentedura et al 2011 [32]	n = 8	n = 1	Greater SE reported in
	Increased neck pain	Increased neck pain	TSM group than cervical
	Fatigue		manipulation group.
	Headache		
	Upper back pain		Duration of symptoms <24
			hours.
Salom-Moreno et al 2014 [29]	n = 1		Duration of symptoms <12
	Cervicothoracic discomfort		hours.
Sillevis et al 2010 [47]	None mentioned	None mentioned	None mentioned
Suvarnnato et al 2013 [30]	None mentioned	None mentioned	None mentioned

Abbreviations: TSM, thoracic spine manipulation

*Adverse effects, AE, are defined as the sequelae following intervention that are medium to long term in duration, with moderate to severe symptoms, and of a nature that is serious, distressing, and unacceptable to the patient and requires further treatment [19]. None of the included studies documented any adverse effects, therefore, there is no data presented in this table.

^†^Un-wanted side effects, SE, are defined as the sequelae following intervention that are short term, mild in nature, non-serious, transient, and reversible [19].

^‡^None reported, The authors of the studies indicated that no participants reported any adverse events or un-wanted side effects.

^§^None mentioned, The authors of the studies did not report on adverse events or un-wanted side effects.

### GRADE evidence profile

A formal grading of evidence was conducted using the GRADEpro software to provide an overall level of evidence for thoracic manipulation in the management of MNP. Four comparisons were assessed: thoracic manipulation versus placebo thoracic manipulation, thoracic manipulation versus mobilization, thoracic manipulation versus standard care, and thoracic manipulation versus cervical manipulation. The overall level of evidence (certainty) ranged from very low to moderate and is presented in Table 6. While there is a growing body of literature supporting the use of TSM, the overall quality of evidence is very low due to downgrading of the RCTs for at least three of the domains (risk of bias, inconsistency, indirectness, imprecision, and publication bias) of certainty assessed with the GRADE approach, suggesting that any estimate of the treatment effect is very uncertain.

**Table 6:** GRADE evidence profile.

Outcome (n = studies)	Follow-up	Risk of bias	Inconsistency	Indirectness	Imprecision	Publication bias	Level of evidence
**TSM versus placebo TSM** (n = 2)							
**Pain** (n = 2)	< 1 week	Not serious	Very serious^a^^,^^b^	Not serious	Very serious^c^	None	⨁OOO VERY LOW
**TSM versus mobilization** (n = 4)							
**Pain** (n = 4)	< 3 months	Not serious	Very serious^a^^,^^c^^,^^d^	Not serious	Serious^c^^,^^e^	None	⨁OOO VERY LOW
**Disability** (n = 2)	< 3 months	Not serious	Not serious	Not serious	Serious^e^	None	⨁⨁⨁O MODERATE
**TSM versus standard care** (n = 6)							
**Pain** (n = 6)	≥ 1 week to ≤ 3 months	Very serious^f^	Very serious^a^^.^^b^^.^^d^^,^^g^	Very serious^g^	Very serious^c^	None	⨁OOO VERY LOW
**Pain** (n = 2)	> 3 months	Serious^h^	Very serious^a^^,^^b^^,^^d^^,^^g^	Very serious^g^	Not serious	None	⨁OOO VERY LOW
**Disability** (n = 6)	≥ 1 week to ≤ 3 months	Very serious^f^	Very serious^a^^,^^b^^,^^d^^,^^g^	Very serious^g^	Very serious^c^	None	⨁OOO VERY LOW
**Disability** (n = 2)	> 3 months	Serious^h^	Not serious	Very serious^g^	Not serious	None	⨁OOO VERY LOW
**TSM versus cervical manipulation** (n = 2)							
**Pain** (n = 2)	≤ 1 week	Not serious	Very serious^a^^,^^b^^,^^i^	Not serious	Very serious^c^^,^^e^	None	⨁OOO VERY LOW

^a^ Studies demonstrated variability in results.

^b^ Symptom duration across studies is variable.

^c^ Studies contained small sample sizes.

^d^ Dosage of intervention varied across studies.

^e^ Studies have wide confidence intervals.

^f^ Studies demonstrated risk of bias associated with performance bias, attrition bias, and selection bias

^g^ Interventions of standard care varied across studies, including general exercise, specific exercise, and modalities

^h^ Studies demonstrated risk of bias associated with performance bias and selection bias

^i^ One study utilized specific cervical manipulation

## Discussion

This systematic review concluded TSM to be more beneficial, without any adverse events and minimal unwanted side effects, than thoracic mobilization, cervical mobilization, and standard care, but no better than cervical manipulation or placebo TSM to improve pain and disability for the management of individuals with MNP.

The results of this current systematic review are consistent with the findings of previous reviews conducted by Huisman et al [33] and Young et al [34], which also concluded that TSM has short-term clinical benefit when compared to modalities, thoracic mobilization, and exercise, but is not more effective than cervical manipulation (Fig 6). This current systematic review also contributes to the current body of evidence in several ways. First, it provided a more in-depth search strategy utilizing clinical trials.gov that assessed for any grey literature that may not have been published due to non-significant results. This provides a better assessment of potential publication bias, which was included in the summary of findings (Table 6). Second, it assessed for risk of bias using the criteria recommended by the Cochrane Collaboration [35] as opposed to the PEDro scale, which was utilized in the three previous systematic reviews on this topic [4, 33, 34]. Third, it conducted meta-analysis to provide an overall effect size for the benefit of TSM when compared to other interventions, which was not performed in the previous three reviews [4, 33, 34]. Fourth, it was able to provide a direct comparison between TSM and thoracic mobilization, which the Young et al [34] study was unable to do. Fifth, unlike most meta-analyses that provide standard mean differences as an overall pooled effect size, this systematic review calculated mean differences so that MCID of the primary outcome measures could be discussed in relation to patient improvement. Finally, and perhaps most important this systematic review provided an overall level of evidence using the GRADE approach, similar to the systematic review by Young et al [34] and demonstrated an overall level of evidence ranging from very low to moderate. This suggests that while research continues to emerge with the addition of five new RCTs since 2014, the overall quality of this evidence has not changed, demonstrating a need for further high quality research.

As it relates to meta-analysis, this current review was able to specifically assess for the effect of TSM versus placebo TSM for pain (Fig 2), and TSM versus thoracic and cervical mobilization for pain (Fig 3) and disability (Fig 7), both of which the recent Young et al study [34] was unable to provide. In addition, this current review was able to provide a pooled effect size for TSM plus standard care versus standard care alone for both short-term and long-term follow-up periods for pain (Fig 6) and disability (Figs 8 & 9). In terms of clinical significance, MCID (VAS 12mm, NPRS 13 points) were met for pain in the immediate and short term when comparing TSM to mobilization (MD -13.63) and for pain in the short-term when comparing TSM to standard care (MD -13.21). The remainder of the meta-analyses for pain and disability did not demonstrate a clinically significant change based on MCID values. This appears to be consistent with the generally very low overall level of evidence for TSM. Finally, it also included the GROC, as well as any adverse reactions or unwanted side effects as secondary outcome measures. This provides a more comprehensive assessment of the role that TSM has in the management of individuals with MNP.

Recently, the Cochrane Collaboration (2015) [35], has suggested to categorize patients into the following sub-groups based on symptom duration: acute (< 6 weeks), subacute (6–12 weeks), and chronic (> 12 weeks). According to demographic data, four studies [5, 7, 15, 20, 32] assessed patients with acute symptom duration, two studies [5, 7, 13, 15, 20, 31, 32] assessed patients with sub-acute symptom duration, and six studies [3, 6, 22, 29, 30, 47] assessed patients with chronic symptom duration. One study [20] included patients across all sub-groups, and one study [17] did not provide any data on symptom duration.

Clinicians may question the safety of thrust manipulation in the management of patients with MNP. While the perceived risks of TSM appear to be less than those associated with cervical manipulation [19], a recent systematic review by Puentedura et al [51] highlighted the potential for serious adverse events following TSM. This is an important consideration in clinical practice with a recent paper demonstrating physical therapists are more likely to perform TSM than cervical manipulation [52]. That being said, none of the included RCTs in this systematic review demonstrated any adverse events following thrust manipulation to either the cervical or thoracic spine. Only transient unwanted side effects lasting less than 24 hours were documented (Table 5). In the absence of contra-indications to thrust manipulation the results of this systematic review demonstrate the added clinical benefit of TSM for the management of individuals with MNP. However, clinicians should consider the risk-benefit analysis of using TSM given the recent research that demonstrated the potential for serious adverse reactions [51].

This systematic review is another example of how manual therapy, demonstrated better short-term benefit in the management of MNP. While the recent increase in clinical practice guidelines, treatment-based classification systems, and clinical prediction rules [8, 12, 53] regarding OMPT have provided clinicians with enhanced approaches to examination and management strategies, effective short-term outcomes, and cost savings [54–57], OMPT alone may be insufficient to achieve long-term clinical benefit [58]. Studies have shown that manual therapy provides a mechanical stimulus that alters various neurophysiological mechanisms and reflexes [23]. These changes in the neuromusculoskeletal system may provide the clinician an opportunity to tap into aspects of the movement system that are resistant to change and potentially alter ingrained maladaptive movement strategies [58–60]. Therefore, the authors suggest that clinicians initially begin with OMPT interventions, and progress beyond the management of body structure and function impairments to encompass all aspects of the movement system that had been difficult to influence previously [58, 61].

Clinicians often implement a variety of manual therapy techniques in clinical practice, which seems to be in agreement with the included studies in this systematic review. While thoracic manipulation was implemented in all included studies, the dosage of this intervention, as well as the position in which it was performed was not standardized. In addition, the treatment received by the comparison/control group was also not standardized. It ranged from thoracic mobilization, to cervical mobilization, modalities, and exercise. This makes it difficult to truly assess the benefit of TSM as there could possibly be confounding from co-interventions. While, this is common in clinical practice, it could be one factor that explains why there was high heterogeneity in some analyses, as well as an overall level of evidence ranging from very low to moderate. Other explanations specific to each meta-analysis and their included studies can be found in Table 6.

While this study contributes significantly to the current body of literature, it is not without limitations. The main limitation is the heterogeneity of the methodology used by the included studies. This was demonstrated by the high I^2^ statistic in the majority of the meta-analyses, suggesting that varying clinical settings, study designs, and methodologies may obscure important differences in effects that should be accounted for with future research. This heterogeneity may question the overall validity of conducting meta-analysis. However, due to the recent growth in published research on the effectiveness of TSM and a recent survey demonstrating clinical implementation of TSM by physical therapists, [52] the authors consider meta-analysis valuable in providing an overall treatment effect of TSM as it is relevant and may aide in the clinical decision making process in the management of individuals with MNP.

Implementation of the Cochrane Risk of Bias tool revealed potential biases across the 14 included studies, which included the following domains: performance bias (blinding of participants and providers, influence of co-interventions, and compliance with interventions) and attrition bias (missing drop-out data and incomplete intention-to-treat (ITT) analysis). As is true for most intervention type studies in physical therapy, blinding the treating physical therapist, while ideal, is not possible in clinical trials of this nature. Moreover, while some studies provided detailed information about participant blinding and others explicitly excluded patients who had previous experience with thrust manipulation, others did not account for participant knowledge of intervention. Future RCTs implementing treatment interventions should account for the influence of co-interventions, compliance with home exercise programs, and analysis of drop outs using ITT analyses. As it relates to publication bias, the authors included searching clinicaltrials.gov to assess for any grey literature that may not have been published due to non-significant results. In addition funnel plots were generated for each meta-analysis conducted, and consistently demonstrated relative symmetry with the majority of points scattered centrally around the overall estimated effect [62]. It has previously been acknowledged that funnel plots may be unreliable methods for assessing publication bias in meta-analyses with less than 10 included studies [62], and the recent Cochrane handbook suggests against using them for their poor reliability and validity in detecting publication bias [63]. However, in conjunction with the clinicaltrials.gov search, the authors concluded that publication bias was not present as indicated in the GRADE summary of findings table (Table 6).

Additionally, the type of patients included in this systematic review represents another limitation. Recently, the Cochrane Collaboration (2015) [35], has suggested to categorize patients into the following sub-groups based on symptom duration: acute (< 6 weeks), subacute (6–12 weeks), and chronic (> 12 weeks). While the authors originally intended to provide a sub group analysis for patients with acute, subacute, and chronic neck pain, this was not possible due to the heterogeneity of symptom duration in the included studies based on demographic results and inclusion criteria. In addition, the lack of long-term follow-up in all but three of the included studies makes it difficult to assess the long-term clinical benefit of TSM. Future RCTs should directly compare prescriptive versus pragmatic approaches for OMPT management of individuals with MNP, classify patients by symptom duration, and include long-term follow-up with a quality of life outcome measure.

## Conclusion

TSM has been shown to improve short-term pain, disability, and self-perceived rating of change in function without an increase in adverse events or unwanted side effects in individuals with MNP when compared to thoracic mobilization, cervical mobilization, and standard care, but not cervical manipulation or placebo thoracic manipulation. While the findings of this review support the short-term clinical benefit of TSM for reducing pain, clinicians should interpret these findings carefully with an overall quality of evidence ranging from very low to moderate.

## Supporting information

S1 Appendix PRISMA 2009 checklist. (DOC)
